# Obstetrics and Gynecology

**Published:** 1893-03

**Authors:** 


### OBSTETRICS AND GYNECOLOGY.

EDITED BY DR. VIRGIL O. HARDON.

---

### TREATMENT OF PELVIC SUPPURATION BY VAGINAL DEBRIDEMENT AND ANTISEPTIC DRAINAGE.

Dr. Felix Formento, of New Orleans, recommends the method of La Royenne in cases of pelvic suppuration, phlegmons, pelvic peritonitis, pyosalpinx, etc., which gives a mortality of only one or two per cent. The operative procedures consist, (1) in puncturing the well limited collection by means of a special trocar in the posterior vaginal *cul de sac*, except in rare cases of abscesses pointing between the vagina and bladder; (2) in incising largely and transversely with a metrotome and keeping the cavity dilated until cicatrization. The means of controlling the hemorrhage, which is of rare occurrence, consists of a properly shaped, fine, elastic iodoformed sponge introduced into and compressing the incision in the *cul de sac*. In order to facilitate the operation, Prof. La Royenne invented a special trocar grooved sound. The transverse debridement should be limited to eight or nine centimeters. The finger will be the best instrument to reach multiple cavities. The puncture of the rectum should be avoided by placing a finger in that organ and another in the vagina. The following rules should be observed:

1. After complete anæsthesia and thorough disinfection, the patient lying on her back with her thighs strongly flexed, the purulent cavity should be once more well defined and the position of womb and rectum well made out.

2. An assistant presses down on the abdomen to cause bulging of the cavity on the vagina.

3. The operator plunges his trocar, properly directed, in the center of the posterior *cul de sac*, immediately back of the os uteri.

4. The trocar, after penetrating the cavity, is withdrawn, and through the groove of the canula, held *in situ*, the metrotome is introduced into the abscess. The canula is withdrawn, the metrotome opened to the proper width and withdrawn while opened, thus incising transversely the walls of the abscess and vagina.

A weak antiseptic solution is injected. A proper sponge, well iodoformed and tied with a thread, is introduced into the abscess and left protruding through the vaginal incision. This method, thus followed, is very simple and offers but little danger.—*New Orleans Medical Journal.*

---

### DERMATOL IN GYNECOLOGY.

Asch does not look upon dermatol as a substitute for iodoform, as is done by many, but claims that it occupies a place *sui generis;* for although it may be used where iodoform, for want of something better, was employed heretofore, still it has an entirely different field of usefulness. Dermatol possesses only slight antiseptic properties ; its action in this direction is more due to its drying properties—it keeps the wound aseptic by making it a poor soil for the propagation of germs. It also hastens the healing of the wound under a dry scab, and does not irritate in the slightest degree. Eczemas produced by sublimate, iodoform and other antiseptic dressings are quickly cured by the employment of dermatol. Through its tendency to keep the dressings dry it permits of their retention longer than could otherwise be done, this also facilitating the healing process. It has the same drying power that is possessed by bismuth. The latter, however, has been found to be poisonous when used in large quantities ; experiments of Heinz and Liebrecht have proven dermatol to be absolutely non-poisonous. Another advantage of the drug is that the powder and the dressings impregnated with it may be sterilized. Neither dry heat nor steam affects it. In this respect it has advantages over iodoform, besides being entirely free from odor. In cases of trachelorrhaphy, dusting the powder on the crevix and packing with dermatol gauze gives more satisfactory results. Its effects, however, are best demonstrated in cases of recent perineal rupture and in perineoplastic operations. It protects the wound and the sutures, preventing these being moistened and soiled. In laparotomies, dusting the wound with the powder permits of longer retention of the sutures than would otherwise be permissible, for they are kept perfectly dry. He strongly recommends dermatol gauze for vaginal tamponing, and states that it quickly cures vaginal catarrh. The powder dusted on erosions also effects a rapid cure.—*Ex.*

---

### A STUDY OF THE INFLUENCE OF TREATMENT OF A SYPHILITIC MOTHER, ESPECIALLY DURING PREGNANCY, UPON THE HEALTH OF THE INFANT.

The author devotes time and care to the study of thirty-two cases of pregnancy in syphilitic women, and reaches the following conclusions:

1. The mortality of the fetus, in cases where the mother has never been under treatment, is enormous, reaching a per cent. of 95.5.

If treatment be applied throughout pregnancy, we may hope to obtain almost complete immunity from this infant mortality.

2. Syphilis attacks the fetus especially during the fifth, sixth, and seventh months of intra-uterine existence.

3. Paternal syphilis is less injurious to the fetus than maternal syphilis.

4. The prognosis differs according to the stage of pregnancy when the mother becomes contaminated.

(*a*) If infection occurs during the first three months and is not subjected to treatment, the mortality during the first few days after delivery reaches one hundred per cent.

(*b*) The prognosis is a trifle better if infection occurs during the fourth and fifth months.

(*c*) In one case of contamination at the eight month, the child lived and was apparently healthy; in a second similar case the child was in good condition at birth, but developed symptoms of syphilis later.

(*d*) Whenever suitable treatment was administered, the mortality was *nil.*

5. Etienne in no case met with any unfavorable results from internal medication during pregnancy.—*Geo. Etienne, in Annales de Gynecol.*

---

### SYMPHYSEOTOMY.

According to the New York correspondent of the *Medical and Surgical Reporter*, Dr. W. T. Lusk performed symphyseotomy on a patient at the Emergency Hospital a short time ago. The woman had been in labor thirty hours when brought to the hospital. An attempt had been made by an outside physician to deliver the child with forceps, which resulted in severe lacerations of the vagina and cervix. The degree of pelvic contraction was such as to forbid any attempt to deliver the child without the operation. As the condition of the mother would not permit of the severe mutilation necessitated by Cæsarean section, the choice lay between craniotomy and symphyseotomy. The latter was chosen as it presented the advantage of the delivery of a living child and involved the mother in less risk from sepsis. After the pubes were shaved and rendered aseptic, an incision was mode through the mons veneris and the symphysis divided from below upward. Assistants held the sides of the pelvis, not allowing a separation of the pubic bones of more than an inch, thus avoiding damage of the sacro-iliac synchondrosis and the pelvic viscera. The child was then delivered by the forceps. The pubes were brought together and held in place by sutures through the soft parts and a tight bandage around the pelvis. At the present writing the child is doing well and the mother is living. But the length of time she was in labor, the severe lacerations she received before admission and her critical condition when an operation was decided upon, give her but slight chance for recovery.—*Ex.*

---

### RAPID DILATATION OF THE UTERUS FOR THE TREATMENT OF HEMORRHAGE.

In a paper read before the Medical Society of London, March 28, 1892, Dr. A. Routh presented the following conclusions :

1. Menorrhagia, and especially metrorrhagia, constitute an indication for dilatation.
2. This is best done by graduated bougies under an anæsthetic.
3. Pyrexia is not to be feared if the procedure is carried out under antiseptic precautions, unless malignant disease or tubal disease is present.
4. That even with tubal disease dilatation is not necessarily contraindicated.
5. That dilatation should precede all other operations for the relief of hemorrhage.
6. That dilatation often suffices to effect a cure.—*Medical Press and Circular.*

